# A Closer Look into the Association between the Sacroiliac Joint and Low Back Pain

**DOI:** 10.51894/001c.21971

**Published:** 2021-04-13

**Authors:** Ashley Wieczorek, Erin Campau, Elizabeth Pionk, Molly E. Gabriel-Champine, Carlos F. Ríos-Bedoya

**Affiliations:** 1 Family Medicine McLaren Bay Region; 2 Division of Scholarly Inquiry McLaren Health Care

**Keywords:** osteopathic manipulative treatment, sacroiliac joint dysfunction, low back pain

## Abstract

**INTRODUCTION:**

Low back pain is the most common type of global disability and annually costs the United States over two billion dollars. Opioids have been used to reduce low back pain, although current evidence concerning efficacy is lacking. Sacroiliac joint dysfunction (SIJD) is estimated to be a primary pain source of low back pain in between 10 and 25% of affected patients. The primary objective of this study was to evaluate the rate of SIJD identified through osteopathic techniques in a convenience sample of patients seeking low back pain treatment. The secondary objective was to assess prevalence of low back pain and SIJD among different age groups, and genders.

**METHODS:**

Retrospective chart reviews were completed the adult patients who had received osteopathic manipulative treatment for low back pain at Family Health and Wellness Center in Essexville, MI from January 2018 through June 2019. The prevalence of patients with SIJD was identified during reviews of osteopathic procedural documentation for patients seeking low back pain treatment. Data regarding patients’ age, sex, and treatment modalities were also extracted. Descriptive statistics consisting of frequencies and percentages were calculated.

**RESULTS:**

A total of 84 patient records were reviewed. A total of 51 (60.7%) patients seeking low back pain treatment were diagnosed with SIJD identified by osteopathic providers. This included patients with both lumbar and sacral diagnoses simultaneously. SIJD alone accounted for 26 (31%) of patients seeking treatment. Female patients were more likely to have SIJD involvement than males. Forty one (48.8%) treated patients were between 45-64 years old. Muscle Energy Technique was documented to be the most used for 68 (81%) patients. In addition, techniques tended to move from direct to indirect for older patients.

**DISCUSSION:**

Our study demonstrated that SIJD appeared to contribute to low back pain in 51 (60.7%) of low back pain cases identified using osteopathic techniques. This is much greater than the previously reported percentages of 10 to 25%. One possible confounding influence included varied resident screening and reporting of sacral dysfunction. Since multiple areas of the body can be treated at one time, our current procedure notes did not allow for distinguishing between which types of modalities were used on each region or capture residents’ preferred treatments.

**CONCLUSIONS:**

Although further studies are needed, our results suggest that knowledge of SIJD’s impact on low back pain could lead to improved patient outcomes such as decreased medical costs and opioid use.

## INTRODUCTION

Low back pain is the fifth most common chief complaint in the primary care setting, affecting more than 26 million Americans.^1^ It has also been estimated that 65-80% of adults will have low back pain during their lifetime.^1,2^ In 2015, a systematic review demonstrated that lower back and neck pain was the single largest cause of US and global disability from musculoskeletal disorders.^3^ Globally, it was the most common reason for disability for persons aged 25 to 64 and the second most common cause in adults between 20 to 24 and 65 to 79 years old.^3^

In addition to the physical toll of low back pain in the US, this high rate of this condition results in a substantial financial impact.^4^ A recent article analyzing health care utilization in an opiate-naive patient population found a 12-month post-diagnosis cost of over $2.5 billion. Non-surgical patients accounted for 70.8%, costing the healthcare system $1.8 billion (i.e., approximately $795 per patient).^4^ Current guidelines recommend against obtaining imaging of the spine within 30 days of a low back pain diagnosis or without a trial of physical therapy.^4^ One-third of patients with low back pain who were treated non-surgically received imaging within 30 days of diagnosis. These patients expended two times greater healthcare dollars than those who followed guidelines and did not receive early imaging.^4^

In contrast, surgical patients in this same study comprised 29.3%, spending $784 million (i.e., approx. $25,613 per patient).^4^ When providers consider surgical low back pain patients, it is important to consider postoperative failure risks. Patients may experience post-lumbar laminectomy syndrome, or failed back surgery syndrome (FBSS) with persistent pain and functional compromise. Unfortunately, as many as 80,000 surgical patients, or roughly 20-40% of the low back surgery population, result in FBSS.^5,6^

Although evidence appears to be lacking in regard to opioids providing short-term low back pain relief, adherence to an opioid regimen has been shown to provide some functional improvement.^5,7^ However, there is an increasing problem with opioid use and dependence in the US. In 2010, 20% of 164 million pain visits were treated with an opioid; meaning, approximately one in five noncancerous pain patients had been prescribed an opioid.^2,8^ This ratio has only been increasing. By 2016, there were 67 opioid prescriptions filled for every 100 Americans with noncancerous pain.^2^ Of more concern, one in four patients receiving long-term opioid treatment for chronic pain have been shown to struggle with opioid use disorder and approximately 130 Americans die every day from an opioid overdose.^8^

One modality with the potential to mitigate opioid use by decreasing low back pain is osteopathic manipulative treatment (OMT). Manipulation is known for its noninvasive, low-risk benefits, and has been demonstrated as a first-line treatment for chronic low back pain.^9–11^ OMT has been defined as, "the therapeutic application of manually guided forces by an osteopathic physician to improve physiologic function and/or support homeostasis that has been altered by somatic dysfunction."^12^ Somatic dysfunction is a malfunction of the body system, which may involve muscles, skeleton, nervous system, and/or lymphatics that leads to overall dysfunction manifesting as pain or impairment. Somatic dysfunction has been shown to be treatable using OMT.^9,12,13^

Osteopathic physicians are trained to look at structure and function as a whole; they connect somatic dysfunctions to patients' symptoms.^14^ Osteopathic physicians have also been specifically taught that there are six main dysfunctions that can be associated with low back pain. One of those dysfunctions is found in the sacroiliac joint (SIJ), seen as restriction of movement at the sacral base.^14^ Previous research has estimated that 10 to 25% of chronic low back pain has a SIJ pain source.^11,15–17^ Interestingly, there is a higher rate (i.e., up to 40%) of SIJD being the source of low back pain in patients with FBSS with the rate in some studies noted as high as 63%.^16–18^

When SIJ is included in the differential diagnosis for chronic low back pain, there is a decrease in not only pain, but in health care expenditures.^10,14,18^ The diagnosis of SIJD can be formulated in several different ways. There are many provocative tests that aid in diagnosis; Flexion Abduction External Rotation (FABER), a stress maneuver that detects hip and sacroiliac joint pathology.^15^ FABER is found to be the most reliable as well as most prevalent in studies examining SIJ pain.^15,19^ The current “gold standard” for SIJ diagnosis and treatment is performing injections with a corticosteroid or anesthetic drug under fluoroscopic guidance to obtain between 50 to 75% pain relief.^10,15,16,18^

Research is currently lacking in osteopathic manipulative diagnosis of low back pain, and SIJD. Recent studies have shown that manipulative treatments have a positive effect on decreasing patients' pain with some improvement on functional status.^9^ By incorporating osteopathic manipulation into patients' treatment plans, there could be fewer costly surgeries and FBSS risks. Improving patients’ function and level of pain could also result in fewer opioid prescriptions for chronic low back pain.

### Study Purpose

The primary purpose of this retrospective descriptive correlational study was to evaluate the relationship between low back pain and sacroiliac joint dysfunction as identified using OMT within the primary care population. Secondarily, this study sought to assess the prevalence of low back pain and sacroiliac dysfunction by age and gender. Before the study, the authors hypothesized that sacroiliac joint dysfunction would be more common than previously found.

## METHODS

The authors’ institutional review board approved the study protocol prior to any data collection. This study was conducted at an outpatient family medicine residency clinic in Essexville, Michigan. Charts were identified and evaluated of patients who were: 18 years and older and had received OMT for low back pain between January 1, 2018 and June 30, 2019.

The McLaren Medical Group’s Business Info Specialist and Physician Biller generated a report based on office billing codes of patients who had a diagnosis of low back pain along with OMT procedure codes. This report listed the patient's name, date of birth and date of service, identifying which charts would be utilized. A report of eligible charts was shredded after use, and all necessary research data was kept on a spreadsheet containing only a unique patient identifier with all study information.

All data were solely recorded on the encrypted McLaren Cloud information system. Specifically, charts were evaluated for the percentage rates of patients with sacroiliac joint dysfunction, as documented note for patients who had received OMT for low back pain. As part of routine OMT procedures for the treatment of low back pain, providers had completed notes of any sacroiliac joint dysfunction diagnosed as based on Osteopathic Manipulative Medicine (OMM) principles.^14^

Patients’ charts were excluded if they were found to be non-English speaking without an interpreter present during treatments, pregnant, or have known mental and/or physical disabilities. There were no vulnerable populations identified for recruitment in this retrospective chart review.

## STATISTICAL ANALYSIS

Before proceeding with statistical analyses, data were examined for data outliers, out of range values, and the need for data cleaning and editing before performing a series of frequencies, proportions, descriptive statistics (e.g., mean, median, and standard deviation) and figures (e.g., histograms and box and whisker plots). After this process was performed, any needed data editing for simplification and clarity was conducted. Descriptive statistics such as percentages were presented from this study. The last author CFR-B performed all statistical analysis using the Stata statistical software package (Stata Corporation, College Station, TX).

## RESULTS

**Figure 1:**
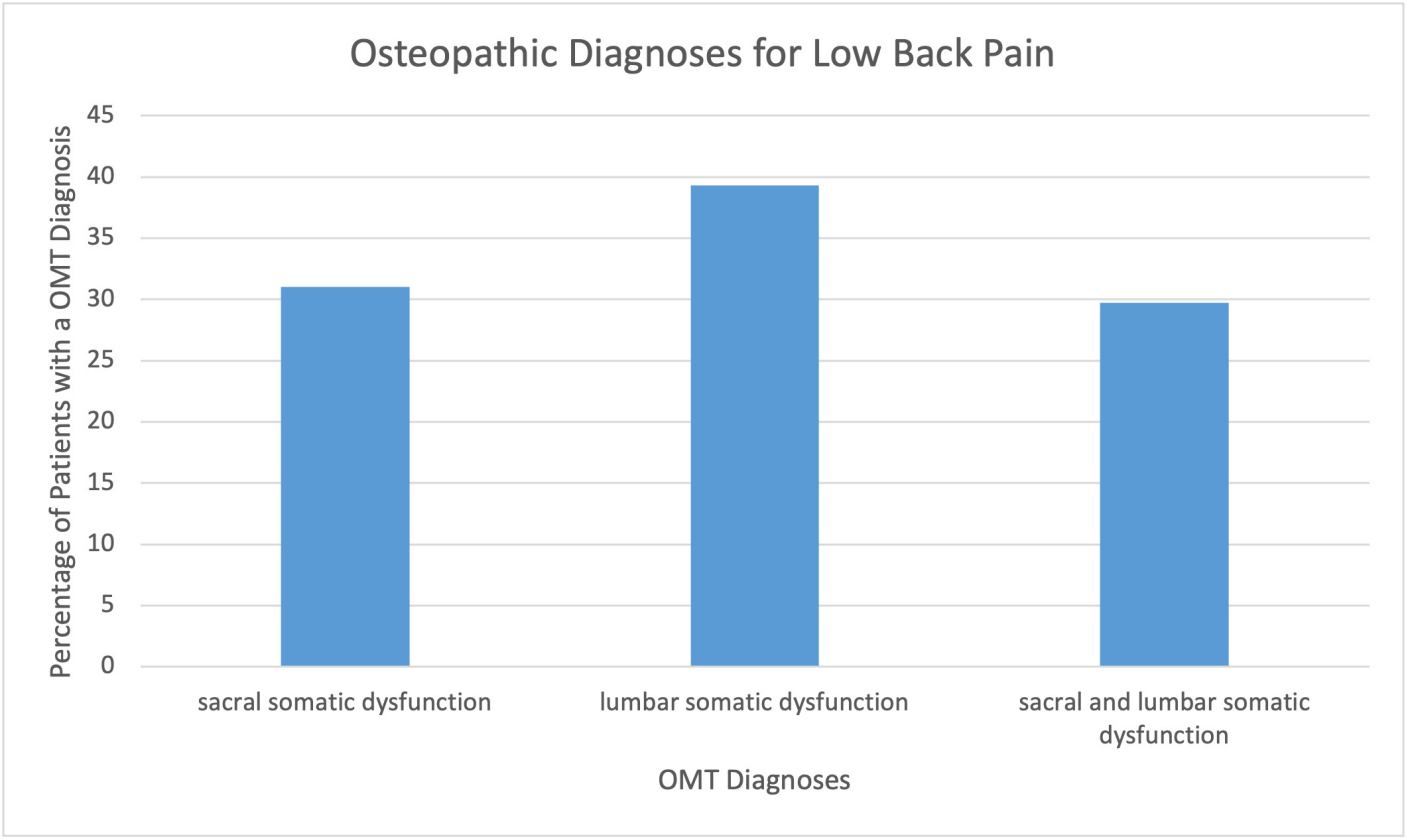
Osteopathic Diagnoses for Low Back Pain

A total of 84 unique patients were identified and reviewed during this study, including 58 (69.0%) females and 26 (31.0%) males. Forty one (48.8%) sample patients who received treatment were between the ages of 45-64 years old. (Table 1) A total of 51 (60.7%) patients who were seeking low back pain treatment were diagnosed with SIJD identified using osteopathic techniques. This number included patients with both lumbar and sacral diagnoses. Sacral diagnoses alone accounted for 26 (31.0%) of patients seeking treatment for low back pain. (Figure 1)

**Table 1:** Patient Characteristics

	**Individuals**	**Percentage**
**Total**	84	100.0%
		
**Gender**		
*Female*	58	69.0%
*Males*	26	31.0%
		
**Age**		
*18-24 years*	7	8.3%
*25-44 years*	33	39.3%
*45-64 years*	41	48.8%
*65+ years*	3	3.8%

The prevalence of somatic dysfunctions were identified overall for patients with a chief complaint of back pain. Lumbar somatic dysfunction alone was most common at 33 (39.3%), followed by only sacral somatic dysfunction 26 (31.0%), then combined sacral and lumbar somatic dysfunction 25 (29.7%). (Figure 1)

**Figure 2:**
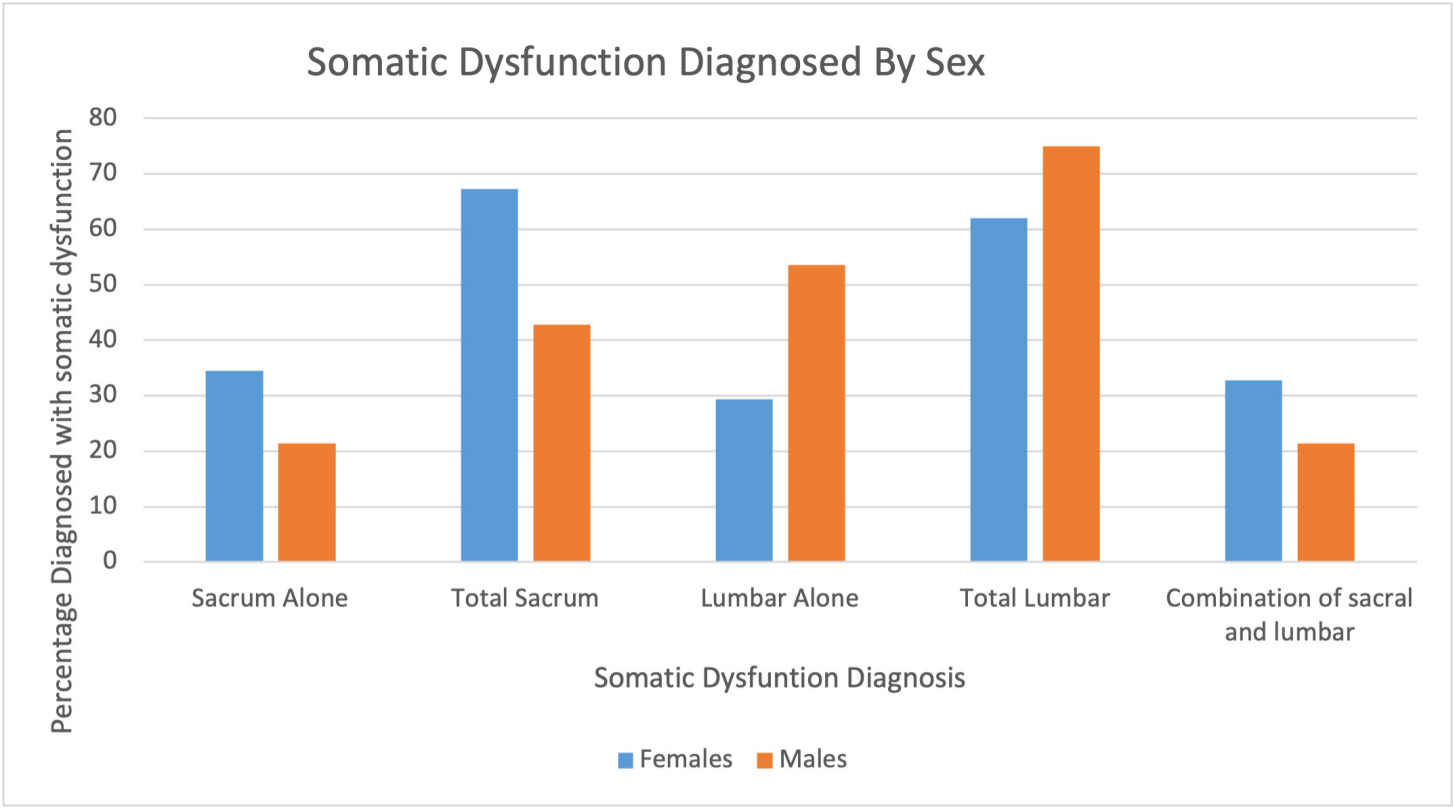
Somatic Dysfunction Diagnosis by Sex.

Our secondary objective was to examine the prevalence of low back pain by gender and explore different treatment modalities used for low back pain. Generally, females were more likely to have SIJD involvement than males. Females had 39 (67.2%) of overall sacral involvement, with 20 (34.5%) females accounting for SIJD alone. While males accounted for 12 (46.2%) sacral or sacral and lumbar involvement and six (23.1%) of sacral alone. (Figure 2)

**Figure 3:**
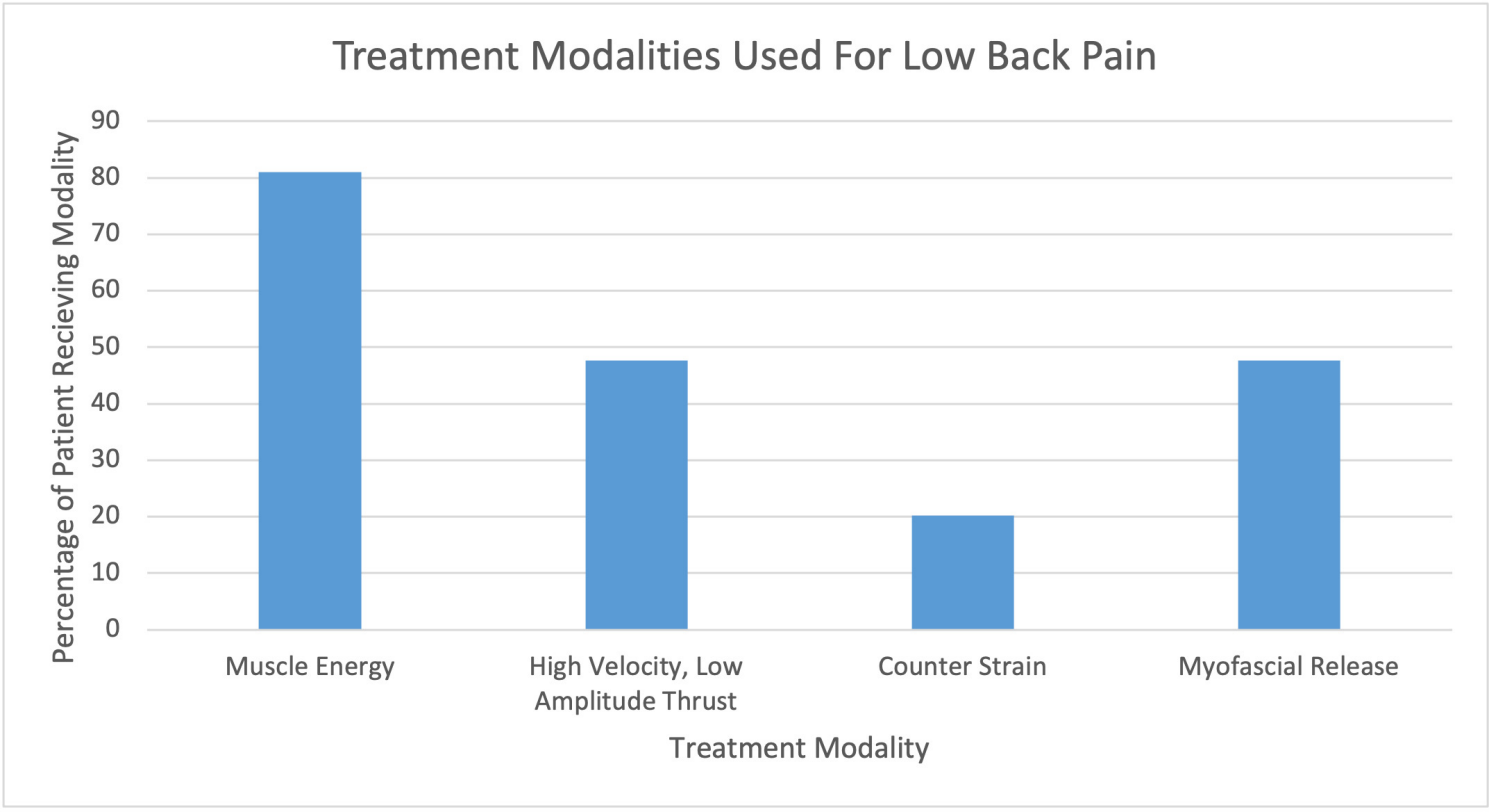
Low Back Pain Treatment Modalities

Although multiple modalities can be individually or simultaneously used to treat low back pain, Muscle Energy Technique (MET) was found to be the most commonly used 68 (81.0%) treatment. Following MET, High Velocity Low Amplitude (HVLA) and Myofascial Release (MFR) were both next at 40 (47.6%). Counterstrain (CS) was used least frequently at 17 (20.2%). (Figure 3)

**Figure 4:**
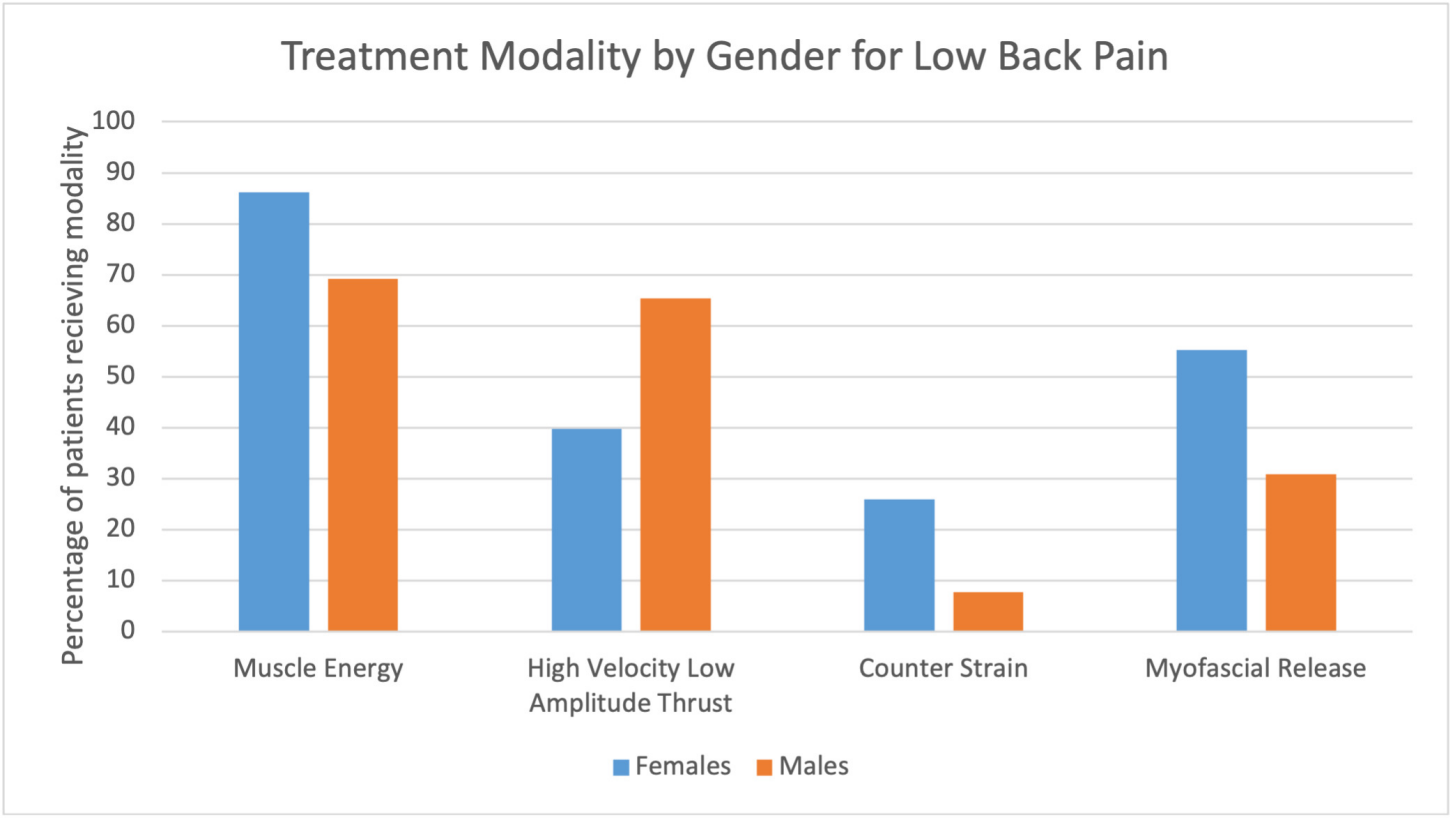
Treatment Modalities by Gender

When examining which modalities were most commonly used for each gender, MET was still the most common for in both female 50 (86.2%) and male 18 (69.2%) patients. Overall, females were more likely to receive treatment multiple modalities. Specifically, females were more likely to receive all types of treatment except for HVLA. For females, MET was most used for 50 (86.2%), followed by MFR 32 (55.2%), HVLA 23 (39.7%), and finally CS 15 (25.9%). For males, MET was most used for 18 (69.2%), followed by HVLA 17 (65.4%), MFR eight (30.8%), and CS two (7.7%). (Figure 4)

**Figure 5:**
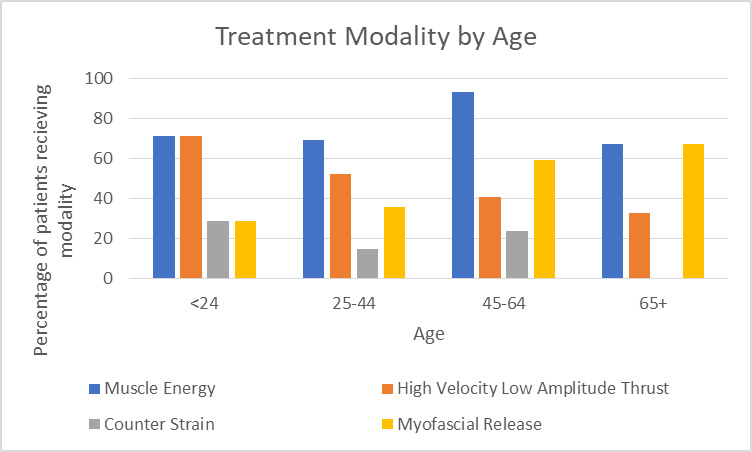
Treatment Modalites by Patient Age

When examining modalities by age subgroup, techniques were seen to change from direct to indirect for older patients. For patients under 24 years old, HVLA and MET were equally used, both being used in five (71.4%) patients in this age subgroup. For ages 25-44, MET was most common (n = 23 (69.7%) with HVLA being second most common at 17 (51.5%). For ages 45-64, MET was again most common (n = 38 (92.7%) with MFR being second most likely at 24 (58.5%) respectively. For patients aged 65 and older, MET and MFR were seen to be equally as common, with both being used in two (66.7%) patients in this age range. (Figure 5)

## DISCUSSION

It is evident from these results that SIJD involvement in low back pain is possibly being overlooked. Our study demonstrated more than double the rate of SIJD than was found in previous studies.^11,15–17^ There are a several possible explanations to explain this finding.

First, our sample size was a small section of the overall low back pain population. Rates of SIJD could potentially be different in a larger sample. Also, many sample patients may have come in for routine treatments of their chronic ailments and may have not always received the same OMT diagnoses during each visit. Incorporating data from each visit may have increased our total sample size. Another explanation for this finding is that osteopathic physicians are trained to seek out other diagnoses which cause low back pain. This could have potentially led to surveillance bias.

There were several confounding influences that may have affected our final results, the largest involving screening and documentation. As our data collection used chart reviews, there was no standardized process for screening patients’ somatic dysfunctions or documentation. Although osteopathic providers are taught systematic ways to screen and document osteopathic findings, these practice patterns are always utilized in practice.

Physicians may not screen for dysfunctions outside of the immediate area, such as in the sacrum, when they are examining a patient for low back pain.^14^ Although it is anatomically related to the back, many patients may not experience, or be able to articulate, pain within in the sacrum, encouraging osteopathic physicians to focus directly on the back or on the patient's expressed area of pain or discomfort.

In 2016, the American Osteopathic Association provided guidelines for OMT to assist osteopaths with regards to low back pain in the proper utilization of OMT rather than guidelines for screening.^9^ Currently, there is no best practice guidelines for screening the lumbar spine using OMT. Furthermore, current healthcare documentation systems do not generally distinguish which OMT techniques are used on which areas. Many OMT modalities can be used on just one area of the body, and similarly multiple areas of the body are often treated at one office visit for OMT.

For example, our documentation system only enables osteopaths to denote that MET, HVLA, MFR and CS were used and that lumbar, sacral, and cervical regions were treated. As a result, we were unable to tell which treatments were used on each region of the body and to what affect. This is likely a nationwide issue and has major room for growth in the osteopathic community.

Unlike our retrospective chart review project, larger-sample prospective studies comparing groups given SIJ injections to those receiving osteopathic manipulation, a less invasive technique, could contribute a new gold standard for diagnosing sacroiliac joint dysfunction. Additionally, longitudinal studies could examine how osteopathic treatments over time impact the severity of low back pain, concurrent analgesic and/or opioid use frequency, and subsequent surgical procedure rates. Another prospective study design would be to record pain scales prior to, and following, treatment of patients’ SIJD to further identify how dysfunctions might impact low back pain.

Finally, it will be important to consider the holistic osteopathic tenants when developing projects in this area of research. Non-invasive osteopathic medicine treatment style improve patients’ quality of life from improved structural and functional self-regulating and self-healing has a significant potential to become a mainstay treatment for SIJD.

## CONCLUSION

Previous research indicates that 10 to 25% of chronic low back pain has a SIJ pain source.^11,15–17^ The current gold standard for diagnosis & treatment is performing SIJ injections using a corticosteroid or anesthetic drug under fluoroscopic guidance to obtain 50 to 75% pain relief.^10,15,16,18^ Utilizing osteopathic techniques for diagnosis and treatment, the authors found that SIJD was present in the majority of patients when examined for low back pain. Based on these results, increased medical knowledge concerning SIJD’s impact on low back pain could lead to decreased medical costs and opioid use.

### Disclosures

The overall results of this study were presented as a poster presentation at the Statewide Campus System Poster Day, May 2020.

### Conflicts of Interest

The authors declare no conflict of interest.
